# Multiplex Cell Fate Tracking by Flow Cytometry

**DOI:** 10.3390/mps3030050

**Published:** 2020-07-17

**Authors:** Marta Rodríguez-Martínez, Stephanie A. Hills, John F. X. Diffley, Jesper Q. Svejstrup

**Affiliations:** 1 Mechanisms of Transcription Laboratory, The Francis Crick Institute, 1 Midland Road, London NW1 1AT, UK; 2 Chromosome Replication Laboratory, The Francis Crick Institute, 1 Midland Road, London NW1 1AT, UK; steph.a.hills@gmail.com (S.A.H.); john.diffley@crick.ac.uk (J.F.X.D.)

**Keywords:** BrdU, EdU, fluorescent cell barcoding

## Abstract

Measuring differences in cell cycle progression is often essential to understand cell behavior under different conditions, treatments and environmental changes. Cell synchronization is widely used for this purpose, but unfortunately, there are many cases where synchronization is not an option. Many cell lines, patient samples or primary cells cannot be synchronized, and most synchronization methods involve exposing the cells to stress, which makes the method incompatible with the study of stress responses such as DNA damage. The use of dual-pulse labelling using EdU and BrdU can potentially overcome these problems, but the need for individual sample processing may introduce a great variability in the results and their interpretation. Here, we describe a method to analyze cell proliferation and cell cycle progression by double staining with thymidine analogues in combination with fluorescent cell barcoding, which allows one to multiplex the study and reduces the variability due to individual sample staining, reducing also the cost of the experiment.

## 1. Introduction

Assessing cell proliferation and cell cycle distribution is often at the basis of cell behavior studies. One of the most widely used methods to study cell proliferation, and in particular cell cycle progression under different conditions, is flow cytometry. Since it was first used to study cell cycle distribution [1], several methods have been developed to allow the analysis of cell proliferation [2]. The simplest approach is to measure DNA content across the cell population at a single time point [3], alone or in combination with other cell cycle markers, such as cyclins [4,5,6]. However, this “snapshot” method provides limited information about cell cycle kinetics. To overcome this problem, methods based on cell synchronization and release have been widely used [7,8]. Unfortunately, two main problems are encountered when using these techniques: (i) many of these methods rely on chemical or biological agents to achieve synchronization, most of which are stress-inducing, which makes them incompatible with the study of cellular responses to other cellular stresses (such as DNA damage), and (ii) many cell types, such as patient cells, primary cells or several transformed cell lines cannot be synchronized. There is a third approach that partially overcomes this problem: the incorporation of thymidine analogues in a time-lapse manner [9]. This allows an estimation of the progression rate through different phases of the cell cycle. To this end, BrdU and other analogues have been widely used [10,11,12,13,14,15,16]; most recently, EdU [16,17,18,19] and click chemistry, which does not require denaturation of the sample before analysis. However, since only one nucleotide analogue can be used, this technique requires the acquisition of individual samples at each time point of interest, which is not always possible when the amount of sample is limited (i.e., in vivo studies or patient samples). Moreover, the power of single labelling is limited as it can only assess the number of cells in each cell cycle phase at a given time point to infer cell cycle dynamics, while it cannot follow the evolution of specific populations (i.e., it cannot be used to detect from where a certain cell population arose, or where it progressed to, in the time window of study, for example, after a certain treatment). The problems encountered in single labelling can be partially solved by the use of double labelling with EdU and BrdU [20]. However, the necessity to individually stain and analyze samples often introduces a bias in the results that can easily lead to misinterpretation of the data. For example, when studying cell cycle distribution, differences in cell number or dye concentration may result in changes in the position of the G1-phase peak, making it difficult to differentiate these variations from biologically relevant ones.

Here, we describe a protocol to combine the use of BrdU and EdU in combination with fluorescent cell barcoding (FCB) [21,22] and DNA dyes. FCB offers a solution to the variability in sample staining and analysis encountered when using the methods listed above. First, it allows for a multiplexed flow cytometry analysis and introduces a normalization of samples which ensures unbiased comparison and detection of small differences. Second, this approach also reduces the amount of antibody and staining solutions needed, resulting in a high reduction of costs. This method can help overcome some restrictions of the currently available methods for analysis of the cell cycle and cell proliferation.

## 2. Experimental Design

**Figure 1:**
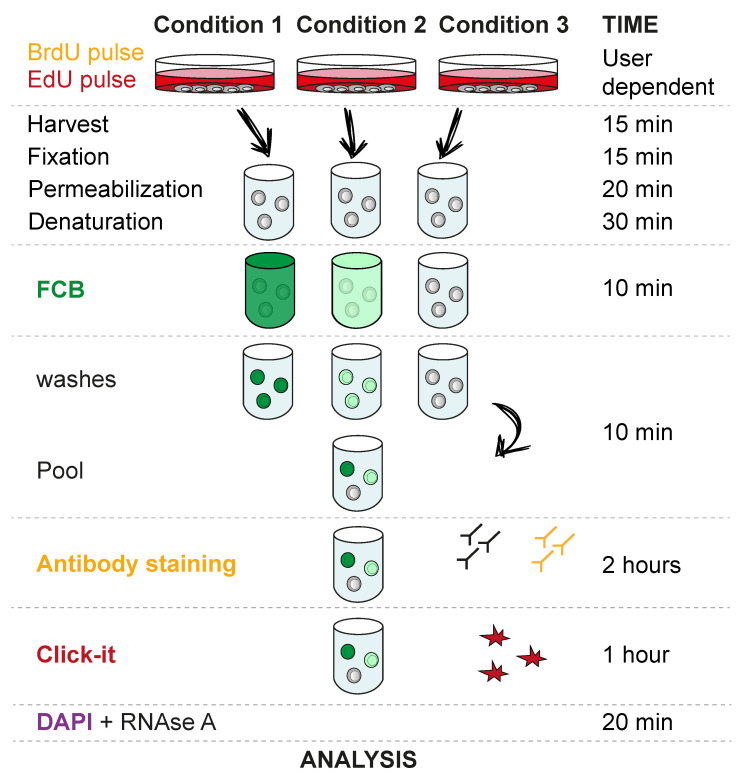
Schematic representation of the method.

An overview of the procedure is shown in Figure 1. This method has been optimized for Flp-In T-REx 293 cells growing in monolayer, but it can be extended to other mammalian cell types. First, cells are labelled with BrdU and EdU following the user’s experimental setup. For example, different time points, cell lines and treatments can be combined to compare the desired parameters in a specific experiment. In this case, the monolayer nature of the culture allows the first analogue to be washed off before labelling with the second analogue, but different approaches can be considered. After harvesting, cells are washed, fixed, permeabilized and denatured, to allow BrdU antibody detection. Then, cells are stained with barcode dyes, allowing the desired populations to be combined and compared. Lastly, unbiased cell staining of the combined population is performed using BrdU antibodies, EdU click-iT chemistry and a DNA dye. The study presented here has been optimized for DAPI staining, but other dyes may be considered.

The success of this method is dependent on the efficient cellular uptake of the thymidine analogue and efficient labelling of newly synthesized DNA. It is therefore critical to check the efficiency of BrdU and EdU incorporation in the specific cell type and experimental setup. It is also advised to check the fluorescent cell barcoding efficiency in the chosen system. A control for the cross-reactivity of the BrdU vs. EdU detection is recommended. Ideally, the control should include two samples, labelled with EdU or BrdU respectively, and combined by FCB to perform the staining procedure on both samples simultaneously. This would allow any cross-reactivity between BrdU and EdU detection methods to be assessed as well as verification of the correct separation of cell populations by FCB. Controls for individual labelling should be included to help define flow cytometry analyzer parameters, as well as compensation, if required.

Interestingly, FCB has been shown to be compatible with fixable viability dyes [22], despite the common nature of the modified molecule, as both are based on amine esterification. This is not included in this protocol, but the use of such dyes may be implemented, if required. The main limitation of the method is that the denaturation step necessary for BrdU staining is not compatible with regular antibody staining.

A scheme summarizing the experimental design, including the time needed to complete every stage, is provided in Figure 1.

### 2.1. Materials

- Flp-In T-Rex 293 Cell Line (Thermo Fisher Scientific, Gloucester, UK; Cat.no.: R78007, RRID:CVCL_U427, authenticated by Thermo Fisher Scientific and routinely confirmed to be mycoplasma-free), or another mammalian cell line or system of choice.
- High glucose DMEM–Dulbecco’s Modified Eagle Medium (Thermo Fisher Scientific, Gloucester, UK; Cat.no.: 11965118).
- Gibco Fetal Bovine Serum (Thermo Fisher Scientific, Gloucester, UK; Cat.no.: 10270098).
- PBS (Phosphate-buffered saline, VWR, Poole, UK; Cat.no.: 45000).
- Trypsin-EDTA Solution 0.25% (Sigma-Aldrich LTD, Gillingham, UK; Cat.no.: T4049.
- Formaldehyde solution 37% (Sigma-Aldrich LTD, UK; Cat.no.: F1635).
- Click-iT™ Plus EdU Alexa Fluor™ 647 Flow Cytometry Assay Kit (Thermo Fisher Scientific, Gloucester, UK; Cat.no.: C10634). A different Alexa dye should not affect the results of the protocol.
- BrdU (5-Bromo-2′-Deoxyuridine, Sigma-Aldrich LTD, Gillingham, UK; Cat.no.: B5002-1G).
- BrdU Monoclonal Antibody (MoBU-1) (Thermo Fisher Scientific, Gloucester, UK; Cat.no.: B35141, RRID:AB_2536441).
- For FBC. Alexa Fluor 488 NHS Ester (Succinimidyl Ester) (Thermo Fisher Scientific, Gloucester, UK; Cat.no.: A20000). A different Alexa dye should not affect the results of the protocol.
- Hydrochloric acid 37% (Sigma-Aldrich LTD, Gillingham, UK; Cat.no.: 258148).
- Bovine serum albumin (BSA) (Sigma-Aldrich LTD, Gillingham, UK; Cat.no.: A3983).
- Ethanol absolute 99.8+% (Thermo Fisher Scientific, Gloucester, UK; Cat.no.: 10437341).
- Goat Anti-Mouse IgG H&L (Alexa Fluor 555) (Abcam, Cambridge, UK; Cat.no.: ab150114, RRID:AB_2687594). A different Alexa dye should not affect the results of the protocol.
- DAPI (4′,6-diamidino-2-phenylindole, Sigma-Aldrich LTD, Gillingham, UK; Cat.no.: D9542).
- RNase A, DNase and protease-free (10 mg/mL) (Thermo Fisher Scientific, Gloucester, UK; Cat.no.: EN0531).

### 2.2. Equipment

- Biological safety cabinet type II
- CO2 incubator
- Chemical fume hood
- Tissue culture plates of the desired size (for instance, Corning, Deeside, UK; Cat.no.: 3506)
- Low binding/maximum recovery 1.5 mL microcentrifuge tubes (for instance, Axigen, Corning, Deeside, UK; Cat.no.: 11311984)
- Swing rotor centrifuge for 1.5 mL microcentrifuge tubes (for instance, Eppendorf, Stevenage, UK; Cat.no.: 5804R with 1.5 ml adapters)
- Falcon 5 mL Round Bottom Polystyrene Test Tube with Cell Strainer Cap (Falcon, Corning, Deeside, UK, Cat.no.: 352235)
- Rotating wheel for 1.5 mL microcentrifuge tubes (for instance, SB3, Cole-Parmer, Saint Neots, UK; Cat.no.: 11496548)
- Flow cytometry analyzer (for instance, LSR II, BD Biosciences, UK)

## 3. Procedure

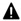
 All centrifugations should be performed in a swing rotor centrifuge at room temperature (RT) to minimize cell loss.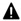 It is critical to pipette up and down after every step to avoid cell aggregation.

All centrifugations are performed at 300 g and RT, 3 min unless stated otherwise.

### 3.1. Cell EdU and BrdU Labelling. Time for Completion: Defined by Experimental Design. 1 Day in This Setup

1. Seed Flp-In T-REx 293 cells in 6 well plates at a density of 5 × 10^5^ cells/well. Add 3 mL of tissue culture medium and leave overnight (O/N) in a CO_2_ incubator at 37 °C and 5% CO_2_.
2. Add EdU and BrdU as required by the experiment. A 30 min 10 µM pulse of each analogue is commonly used for cell cycle studies.

### 3.2. Harvest, Fixation, Permeabilization and Denaturation. Time for Completion: 01:20 h

1. Harvest cells by washing once with PBS and adding 250 µL trypsin to a well of a 6-well plate for 3 min or until cells are fully detached (other harvesting methods may be used, depending on the cell type and experiment setup). Neutralize trypsin by adding 1 mL of tissue culture medium and transfer cells to a 1.5 mL low binding tube. Wash once with PBS.
2. Resuspend in 500 µL of freshly prepared 4% formaldehyde and incubate 10 min at RT in the dark. Wash once with 500 µL wash buffer.
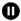
**PAUSE STEP**: After wash, the mix can be stored at 4 °C for up to one week in wash buffer.
3. Resuspend in 500 µL 70% ethanol and incubate 20 min at −20 °C. Wash once with 500 µL wash buffer.
4. Resuspend in 500 µL 2N HCl and incubate 20 min at RT. Wash twice with 500 µL wash buffer.

### 3.3. Fluorescent Cell Barcoding (FCB). Time for Completion: 00:20 h


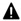
**CRITICAL STEP** Use the previously prepared NHS Ester stock solution to prepare the dilutions indicated in Table 1. Dilutions can be kept at −20 °C for a few weeks. It is important to verify how many dilutions can be used efficiently in the cell type of choice. We have used up to 6 dilutions in non-denaturing conditions. However, after the denaturation step necessary for BrdU staining, we recommend using only 3 dilutions, as indicated in Table 1.

1. Each sample will be stained with one concentration of the dye. To do so, add 3 µL diluted dye to 147 µL wash buffer (70% ethanol can also be used). For the compensation control, the highest concentration is recommended.
2. Add the total 150 µL to the sample and incubate for 10 min at RT.
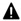
**CRITICAL STEP** Wash 3 × 150 µL wash buffer. It is very important to thoroughly wash the samples to remove any non-incorporated dye before pooling the samples.
4. Pool samples, spin down and remove supernatant.
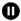
**PAUSE STEP**: Samples can be kept O/N in at 4 °C wash buffer.

### 3.4. BrdU Antibody Staining and EdU Click-iT. Time for Completion: 03:00 h

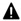
**CRITICAL STEP** Add 200 µL 1:50 BrdU Monoclonal Antibody (MoBU-1) in wash buffer to each sample and incubate 45 min at RT. It is absolutely necessary to use this specific antibody clone (MoBU-1), as it has no cross reactivity with EdU.
2. Wash 3 × 150 µL wash buffer.
3. Add 200 µL 1:200 Goat Anti-Mouse Alexa Fluor (555) in wash buffer to each sample and incubate 45 min at RT.
4. Wash 3 × 150 µL wash buffer.
5. Perform Click-iT reaction following the manufacturer’s instructions.

### 3.5. RNase A Treatment and DAPI Staining. Time for Completion: 00:20 h

1. Resuspend cells in 200 µL wash buffer containing 100 µg/mL RNase A and 1 µg/mL DAPI and incubate for 15 min.
2. Centrifuge and resuspend in 200 µL wash buffer.
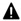
**CRITICAL STEP** Transfer solution to a 5 mL round bottom polystyrene test tube with cell strainer cap. It is necessary to filter the cell suspension through the tube cap to avoid cell clumps.

### 3.6. Analyze in Flow Cytometry Analyzer. Time for Completion: Defined by Experimental Design

- The parameters used in this illustrative example are shown in Table 2; however, others can be used depending on the user requirements and availability.

## 4. Expected Results

The analysis of the samples should show a good separation of the pooled samples by the FCB parameter. It should also show no cross reactivity between BrdU and EdU.

**Figure 2:**
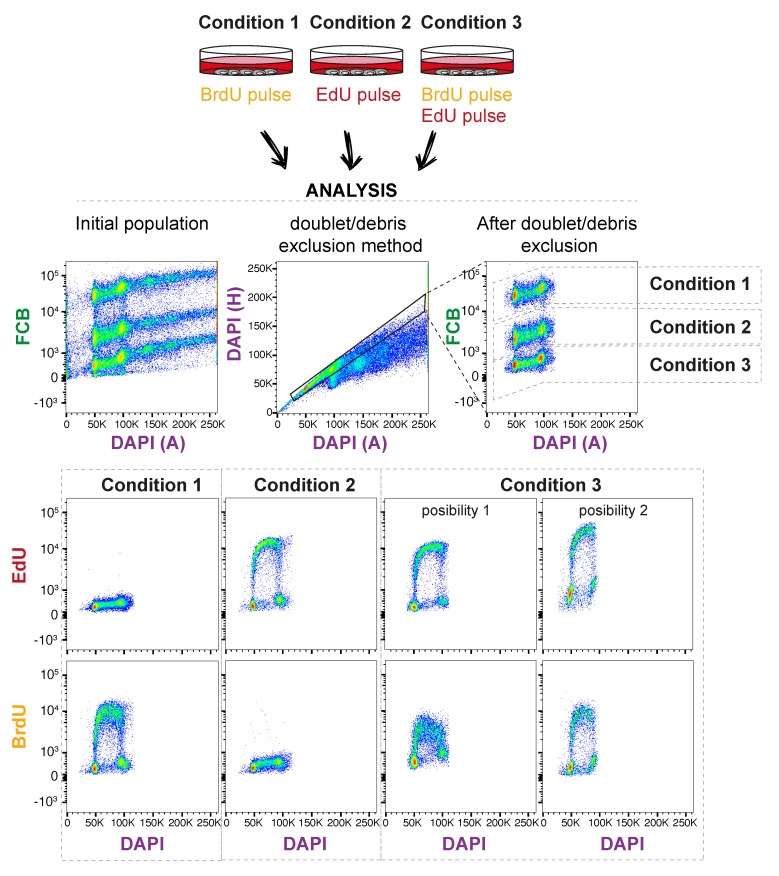
Expected results for the experiment. In this example, three different conditions are used for labelling the cells as indicated by conditions 1 to 3. The analysis performed in FlowJo should look as shown in the figure. Firstly, the populations can be easily distinguished by FCB, even before cell doublets and debris exclusion. Afterwards, gating is used to separate these populations and analyze BrdU and EdU staining. As expected, no cross reactivity is observed between BrdU and EdU (condition 1 and 2). Examples of two possible outcomes are shown for condition 3. In these, the variability in BrdU and BrdU staining are exemplified (note that possibility 2 is from a different experiment). Importantly, this variability does not affect the interpretation of the experiment, as the use of FBC (allowing all samples to be stained in the same tube) ensures that any observed changes are not due to variations in staining. This, and other potential issues arising, and their troubleshooting, are discussed in Table 3. Colors in the flow analysis graphs indicate cell population density, as defined by FlowJo analysis tools.

Figure 2 provides a description of the analysis and results to expect in a successful experiment.

## 5. Reagents Setup

### 5.1. Tissue Culture Medium

- High glucose DMEM
- 10% v/v FBS
- 100 U/mL penicillin
- 100 μg/mL streptomycin

### 5.2. Wash buffer

- PBS
- 1% BSA

### 5.3. NHS Ester Stock Solution

Prepare 1 mg/mL in DMSO

### 5.4. BrdU 100µM

Prepare 10 mM stock (1:1000) in DMSO

## Figures and Tables

**Table 1:** Serial dilution to be done from the 1 mg/mL NHS Ester stock solution.

Final Concentration	Make 50x	Dye (from Previous Dilution)	DMSO
µg/ml	µg/ml	µL	µL
15	750	75	25
5	250	33.3	66.6
1.3	65	26	74
0.3	15	23.1	76.9
0.075	3.75	25	75
0	0	0	100

The three recommended dilutions to use together in the same experiment after denaturing are indicated in either dark or light green (i.e., 15, 1.3 and 0.075 or 5, 0.3 and 0).

**Table 2:** Parameters used in the flow cytometry analyzer LSRII.

Reagent	Laser	Bandpass Filter
FCB (Alexa 488)	488	525/50
BrdU (Alexa 555)	561	582/15
EdU (Alexa 647)	633	660/20
DAPI (UV)	355	450/50

**Table 3:** Potential issues arising and respective troubleshooting.

Issue	Possible Causes	Suggestions
Cell loss	Cell loss during centrifugation	Use swing rotor as recommended
Inefficient FCB detection	Cells had formed aggregates after step 3.2 Too many cells used for labelling Not enough washing before pooling the samples after FCB	Pipette up and down to ensure cell dispersion after 3.2 Do not exceed 4 × 10^6^ cells per FCB dilution (12 × 10^6^ cells total) Increase wash volume and time
Insufficient FCB population separation	Dye is too old Cell type used requires different dilutions	Use freshly prepared dilutions Optimize dilutions prior to use
Poor BrdU detection	Not enough washes after 3.2 Too many cells used for labeling	Increase washing time and volume Do not exceed 12 × 10^6^ cells total
Poor EdU detection	Not enough washes after 3.2 Too many cells used for labeling	Increase washing time and volume Do not exceed 12 × 10^6^ cells total
